# Flow reversal in distal collaterals as a possible mechanism of delayed intraparenchymal hemorrhage after flow diversion treatment of cerebral aneurysms

**DOI:** 10.3389/fphys.2022.881627

**Published:** 2022-07-18

**Authors:** Sara Hadad, Aseem Pradhan, Ramanathan Kadirvel, David Kallmes, Juan R. Cebral, Fernando Mut

**Affiliations:** ^1^ Bioengineering Department, George Mason University, Fairfax, VA, United States; ^2^ Interventional Neuroradiology, Mayo Clinic, Rochester, MN, United States

**Keywords:** delayed intraparenchymal hemorrhage, cerebral aneurysm, flow reversal, mechanism, flow diversion

## Abstract

**Background and Purpose:** Delayed intraparenchymal hemorrhages (DIPHs) are one of the most serious complications of cerebral aneurysm treatment with flow diverters (FD), yet their causes are largely unknown. This study analyzes distal hemodynamic alterations induced by the treatment of intracranial aneurysms with FDs.

**Methods:** A realistic model of the brain arterial network was constructed from MRA images and extended with a constrained constructive optimization technique down to vessel diameters of approximately 50μm . Different variants of the circle of Willis were created by alternatively occluding communicating arteries. Collateral vessels connecting different arterial trees were then added to the model, and a distributed lumped parameter approach was used to model the pulsatile blood flow in the arterial network. The treatment of an ICA aneurysm was modeled by changing the local resistance, flow inertia, and compliance of the aneurysmal segment.

**Results:** The maximum relative change in distal pressure induced by the aneurysm treatment was below 1%. However, for certain combinations of the circle of Willis and distal collateralization, important flow reversals (with a wall shear stress larger than approximately $1.0\;dyne/cm^{2}$ ) were observed in collateral vessels, both ipsilaterally and contralaterally to the treated aneurysm.

**Conclusion:** This study suggests the hypothesis that flow diverters treatment of intracranial aneurysms could cause important flow reversal in distal collaterals. Flow reversal has previously been shown to be pro-inflammatory and pro-atherogenic and could therefore have a detrimental effect on these collateral vessels, and thus could be a suitable explanation of DIPHs, while the small distal pressure increase is not.

## 1 Introduction

Flow diverters (FDs) have been gaining popularity for the treatment of intracranial aneurysms (IAs), especially complex aneurysms difficult to treat with coils or clipping. However, several studies have reported hemorrhagic complications associated with delayed aneurysm rupture or delayed intraparenchymal hemorrhage (DIPH), both with severe consequences and often fatal (Cirillo et al., 2012; Arrese et al., 2013; Brinjikji et al., 2013; Rouchaud et al., 2016). The occurrence of these complications is not uncommon, it has been estimated at 1% for delayed aneurysm rupture (Kulcsar and Szikora, 2012), and 2–3% for DIPH (Rouchaud et al., 2016).

Although the mechanisms of both complications are largely unknown, they likely are quite different from each other since one involves rupture of the treated aneurysm while the other is related to hemorrhages that occur distally from the treated aneurysm. The current study focuses on the DIPHs alone. There are two main characteristics that could help understand the causes of DIPHs. First, although most DIPHs (80%) occur in the ipsilateral vascular territory of the treated aneurysm, others (20%) occur in other territories (Rouchaud et al., 2016). Second, they occur in a delayed fashion, most (86%) within the first month after treatment and some (24%) as early as 24 h after treatment (Rouchaud et al., 2016).

Previous studies have suggested different mechanisms to try to explain these DIPHs, but most of them contradict or fail to explain some of the clinical observations. Embolic events have been proposed as a possible mechanism (Hu et al., 2014), but cannot explain hemorrhages in contralateral territories. Antiplatelet therapy was also suggested in an attempt to explain non-ipsilateral hemorrhages (Rouchaud et al., 2016), but secondary stroke prevention studies suggest that it is relatively safe (Diener et al., 2004), and all territories should be equally affected, not explaining the higher prevalence of ipsilateral hemorrhages. Another conjecture that has been proposed is the hemorrhagic transformation of ischemic lesions caused by the FD aneurysm treatment, but it has not been demonstrated that these lesions co-localize and are therefore the source of the hemorrhages (Tomas et al., 2014). Lastly, hemodynamic alterations induced by the FD implantation have been suggested as possible causes of DIPH. One study investigated the effects of changes in arterial compliance after FD treatment on the distal pressure but found that the resulting pressure increases were insignificant and not sufficient to explain the DIPHs (Mitha et al., 2015). In another report, the authors speculated that changes in hemodynamic stress caused by the stiffening or slight alteration of the parent artery course due to the FD implantation may provide an explanation for the DIPHs (Velat et al., 2012), but the issue was not further investigated.

Thus, the objective of this study was to analyze the distal hemodynamic alterations (including in distal collateral vessels) induced by the treatment of intracranial aneurysms with flow diverting devices, using an extensive computational model of the brain circulation.

## 2 Methods

### 2.1 Modeling the brain vasculature

**FIGURE 1:**
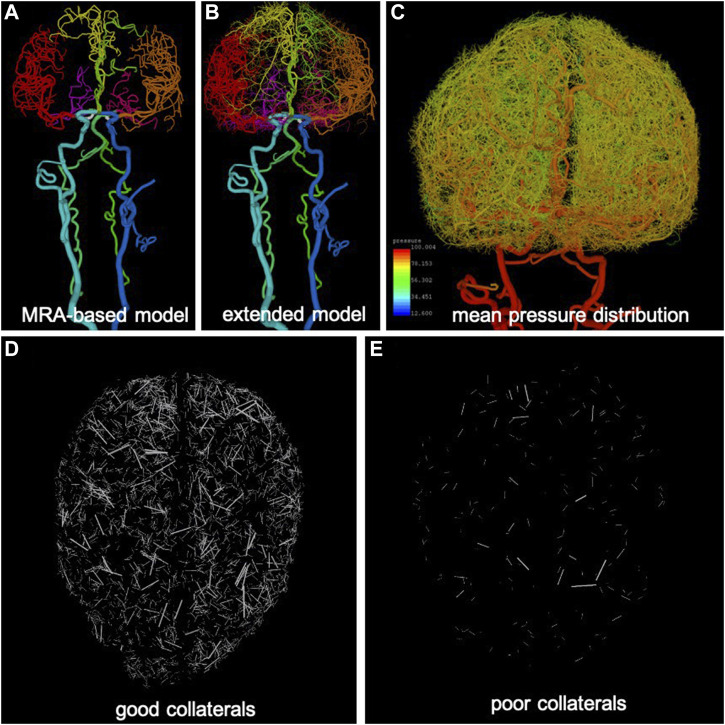
Anatomical model construction: **(A)** arteries reconstructed from MRA images (different arterial trees are rendered with different colors), **(B)** extension of arterial trees using CCO approach (intermediate stage), **(C)** pressure distribution calculated along arterial trees and used for generation of collateral vessels in extended arterial model, **(D)** example of good level of collateralization (superior-inferior view), only collateral branches are visualized, and **(E)** example of poor collateralization.

A detailed computational model of the brain arterial network, spanning from the aortic arch to small cerebral arteries of approximately 50 μm , and including different collateral paths provided by the circle of Willis (primary collaterals) as well as distal collaterals (e.g., pial or lepto-meningeal collateral arteries) was constructed in four stages as detailed below (Figure 1; Supplementary Appendix S1).

#### 2.1.1 Image-based arterial reconstruction

The major vessels feeding the brain, starting with the common carotid arteries (CCAs) and vertebral arteries (VAs), and continuing to the main intracranial arteries (internal carotid arteries—ICAs, basilar artery—BA) and the circle of Willis (posterior communicating arteries—PCOMs, and anterior communicating artery—ACOM) were reconstructed from MRA images of normal subjects using previously developed methods (Cebral et al., 2005). Similarly, the main arterial trees emanating from the circle of Willis and feeding the two brain hemispheres (anterior cerebral artery—ACA, middle cerebral artery—MCA, posterior cerebral artery—PCA) were reconstructed for a few generations down to the image resolution (about 0.5 mm) (Wright et al., 2013). The resulting vascular model contained approximately 3,400 arterial branches (Figure 1A).

#### 2.1.2 Arterial tree extension

**FIGURE 2:**
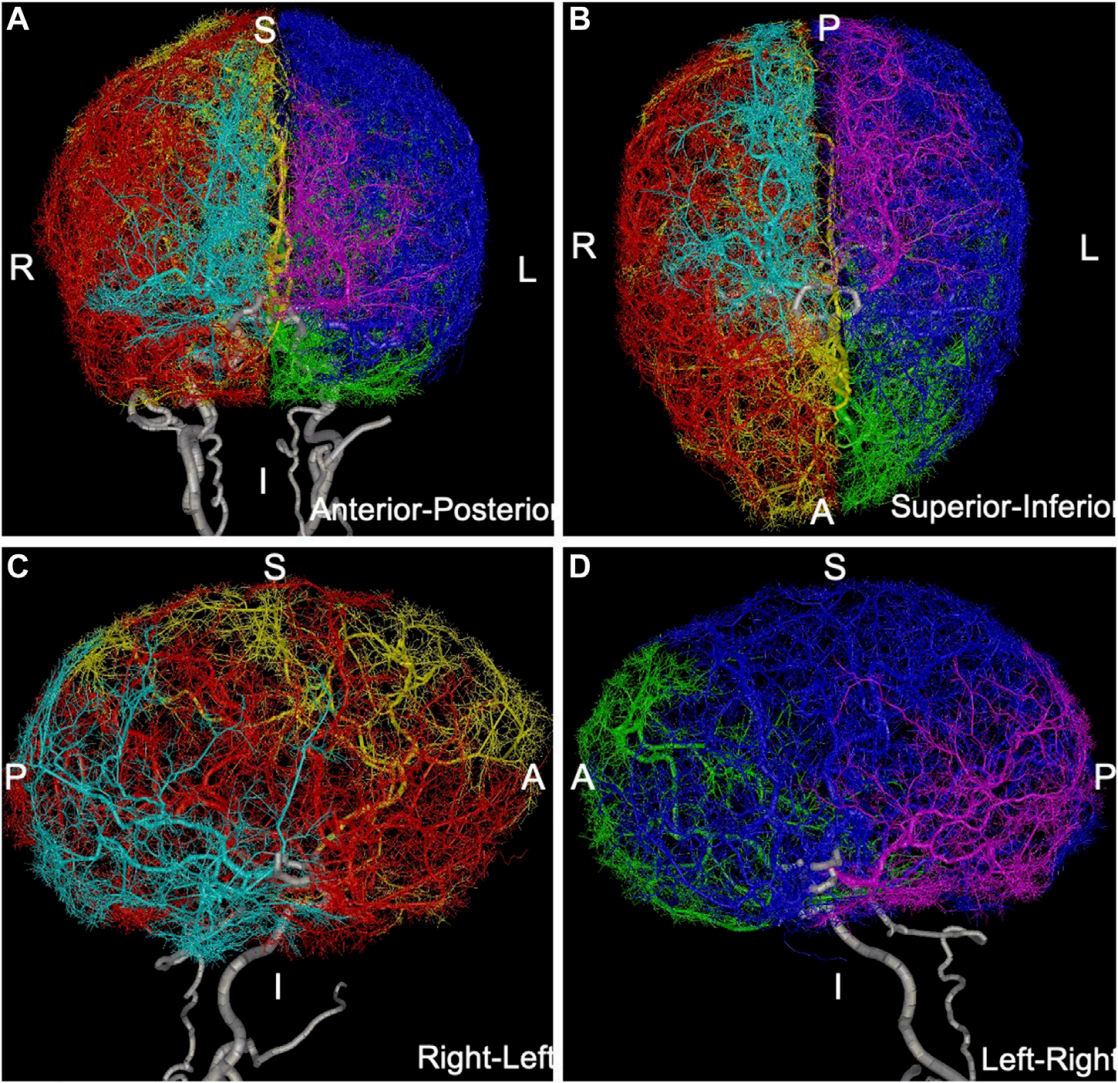
Anatomical model containing over 100,000 arterial branches constructed by extending MRA-based model with CCO method: **(A)** anterior-posterior view, **(B)** superior-inferior view, **(C)** right-left view, **(D)** left-right view. Different arterial trees are rendered with different colors: left-ACA = green, left-MCA = blue, left-PCA = magenta, right-ACA = yellow, right-MCA = red, right-PCA = cyan, main feeding vessels and circle of Willis (ICAs, VAs, BA, PCOMs, ACOM) are rendered in white.

The image-based arterial model resulting from the previous stage was then synthetically extended down to vessel sizes of approximately 50 μm using a constrained constructive optimization (CCO) strategy (Bui et al., 2009). The goal of this approach was to uniformly perfuse a given volume following an optimization criterion, in our case we chose to minimize the total vascular volume (Schreiner et al., 1995). The algorithm took as input the perfusion volume given as a 3D tetrahedral mesh (which provided great flexibility in defining the volume) and an initial set of arterial branches. The perfusion volume was defined as the brain parenchyma, which was created by a rough segmentation of the brain from the MRA images, and subsequently meshed (filled) with tetrahedral elements using an advancing front method (Löhner, 1996). The left and right brain hemispheres were meshed separately, and the three left and right arterial trees (ACA, MCA, PCA) were initialized from the corresponding vascular segmentations. The algorithm then proceeded as follows: starting with the given arterial trees, a point was randomly selected within the perfusion volume (i.e. by randomly picking a point in a random tetrahedral element), and a new terminal branch was created connecting this point to the branch of the tree that resulted in the minimum total vascular volume. Following the principle of minimum work or Murray’s law for arterial bifurcations ( $r_{0}^{3}=r_{1}^{3}+r_{2}^{3}$ , where $r_{0},\;r_{1},\;r_{2}$ are the radii of the parent and daughter branches respectively (Bui et al., 2009)), when a new terminal point was connected to a tree, all branches downstream of the new bifurcation needed to be scaled to accommodate the new unit of flow through the new branch. For each new terminal point, several nearby branches (possibly of different trees) were tried, and the point was connected to the one that results in the minimum total vascular volume, after checking that the new branch did not intersect any existing branch. Note that in this algorithm, all arterial trees were extended simultaneously in a single perfusion volume for each hemisphere which was not partitioned *a priori* into vascular territories, thus the trees “competed” to supply different regions of the brain. As such, the final territory of each tree was determined by the initial vascular reconstructions on the large scale, and by the small-scale competition between adjacent trees in borderline regions. The procedure was repeated independently for each of the brain hemisphere until the desired number of terminal points in each hemisphere was reached (Figure 1B). The extended vascular model contained approximately 100,000 arterial branches and is presented in Figure 2. The procedure is schematically illustrated in Supplementary Figure S1.

#### 2.1.3 Variants of the circle of willis

Anatomical variants of the circle of Willis were generated by alternatively removing (occluding) from the full model each of the communicating arteries in turn (left and right PCOM and ACOM) as well as maintaining the ACOM but removing the A1 segment of the left and right ACA, while keeping the rest of the network intact. This strategy resulted in a total of 16 combinations corresponding to different configurations of the primary collaterals of the circle of Willis (see Supplementary Appendix S2).

#### 2.1.4 Generation of pial collateral vessels

Pial or lepto-meningeal collaterals are small arteries connecting arteriolar branches of different brain arterial trees (Brozici et al., 2003). Their numbers, sizes, and distributions are highly variable among individuals, but they connect branches ranging from about 400 μm to 50 μm , and under normal conditions there is essentially no flow through these collaterals, they are typically recruited under pathological conditions such as vessel occlusions (Brozici et al., 2003; Faber et al., 2014). As such, pial collaterals connecting branches of different arterial trees (i.e., ACA, MCA, PCA) in each brain hemisphere were generated obeying the following criteria: a) they connect branches that are in close proximity to each other, b) they connect branches that are roughly at the same mean arterial pressure (which implies no or very small flow through the collateral under normal conditions), c) they connect branches within a certain range of diameters (e.g. smaller than 400 μm ), d) they do not intersect any other branch, and e) their aspect ratio (length over diameter) does not exceed a given threshold (e.g., L/D<20) . In order to compute the mean pressure along each arterial tree, a lumped parameter model (see next section) was used (Figure 1C). The algorithm then proceeded as follows (see Supplementary Figure S1): randomly pick a branch with diameter in the appropriate range, find close branches of different trees with similar mean pressure, create a new collateral with diameter equal to the average of the diameters of the two branches, accept the collateral if it does not intersect any other branch. Note that with this approach, the new collateral bifurcations do not follow Murray’s law and the downstream trees are not changed, which makes sense since under normal conditions there should be no flow through the collaterals (i.e. as if they were essentially absent). Following this approach, different levels of collateralization of the brain (i.e. different number of pial collaterals) were generated, denoted excellent (n = 6,000), very good (n = 4,000), good (n = 2000), fair (n = 1,000), poor (n = 500), very poor (n = 100), and none (n = 0) collateralization, respectively. The process was repeated for each of the variants of the circle of Willis. Examples are shown in Figures 1D,E.

### 2.2 Modeling blood flows

In order to model the blood flow through the large arterial network described above, a distributed multi-compartment lumped parameter model was used (Peiro and Veneziani, 2009) (see Supplementary Appendix S3 for details). Each arterial branch (including collaterals) was subdivided into a series of connected compartments. Each compartment was characterized by a flow resistance R (accounting for viscous flow effects), an inertance L (accounting for blood inertia effects), and a capacitance C (accounting for vessel wall compliance). These quantities depend on the blood density ( ρ=1.0 g/cm^3 ) and viscosity ( $\mu =0.035\;g/cm^{3}.s$ ), segment length ( l= Euclidean distance between end points of branch segment in vascular model) and undeformed radius ( $r_{0}$ from vascular model), wall elasticity ( $E=3\times 10^{6}\;dyne/cm^{2})$ and thickness ( $h=0.1\times r_{0})$ (Formaggia et al., 2003). These parameters were constant throughout the network (including collaterals) except for the wall thickness which was estimated as 10% of the vessel radius. The unknowns were the pressure P at the nodes connecting compartments (i.e., inlet and outlet of each compartment) and the flow rate Q through each compartment. At arterial bifurcations (or junctions), continuity of pressure and conservation of mass (flow) was applied. The resulting system of equations for the entire arterial network was solved using a sparse matrix linear solver (Davis, 2006).

Boundary conditions were applied as follows (see also Supplementary Appendix S3). At the network inlets (common carotid and vertebral arteries) pulsatile inflow rates were specified as time dependent functions Q(t) (Supplementary Figure S4A). At each of the outlets, a resistor representing the total resistance of the distal vascular bed not included in the model was added. These resistances were estimated according to Murray’s law by splitting the total inlet flow with the outlet area to the 3/2 power. A constant reference (venous) pressure ( $P_{v}\approx 0\;mmHg)$ was prescribed at the resistor outlets. Similar boundary conditions were used for all vascular models with the different collateralizations. The timestep size was set at Δt=0.0001s after verifying convergence as detailed in Supplementary Appendix S3 and illustrated in Supplementary Figure S5.

### 2.3 Modeling aneurysms and flow diversion

The presence of an intracranial aneurysm in the ICA was modeled by changing the local resistance and inertance of the arterial segment corresponding to the aneurysm location. The magnitudes of these changes were obtained from 3D patient-specific models of ICA aneurysms studied before (Mut et al., 2015) using a CFD approach previously described (Mut et al., 2010; Detmer et al., 2020). Briefly, the pressure drop ΔP along the axis of the parent artery was obtained from steady CFD simulations at different inflow rates Q , and the corresponding flow resistance was calculated as the slope of the ΔP vs Q curve (i.e. ΔP=RQ ). These calculations were performed with (original model) and without the aneurysm, i.e. after virtually removing the aneurysm to estimate the change in local resistance ( ΔR ) caused by the presence of the aneurysm (see Supplementary Appendix S4). The process was repeated for 27 different ICA aneurysms and the maximum resistance changes were estimated.

Similarly, the change in local inertance due to the presence of a cerebral aneurysm was estimated by performing unsteady CFD simulations with a sinusoidal inflow waveform and computing the slope of the $\text{Δ}P_{L}\;vs\;\omega q$ curve (see Supplementary Appendix S5). As with the resistance, the maximum relative change in local inertance ( ΔL ) due to the presence of an aneurysm was estimated by computing L with and without the aneurysm for the same 27 ICA aneurysms.

The implantation of a metallic flow diverting device to treat an intracranial aneurysm also results in a local stiffening of the vessel wall, which can be observed as an increase in the pulse wave velocity (Kolumam Parameswaran et al., 2019). Estimates of the change in local compliance C were obtained from experimental measurements of pulse wave velocity obtained before and after deployment of flow diverters in the aorta of rabbit models in a previous study (Kolumam Parameswaran et al., 2019). Specifically, that study showed an average increase in pulse wave velocity from 0.37±1.09 m/s to 1.18±0.54 m/s , i.e., an increase by a factor of approximately 3.18 after FD deployment (Kolumam Parameswaran et al., 2019). Assuming a linear elastic material, this change translates into an increase in arterial elastic modulus by a factor of approximately 10.17 (elasticity modulus is proportional to the square of the pulse wave velocity). The same study found that the arterial segment immediately distal to the flow diverter was also similarly stiffened at the follow up time after treatment.

### 2.4 Modeling different scenarios

In order to model the flow conditions before and after treatment of a cerebral aneurysm with a flow diverter, two scenarios were created: a) pre-treatment: the local flow resistance R and inertance L were changed at the aneurysm location to account for the presence of the aneurysm, and b) post-treatment: the aneurysm was removed (reverted to the original R and L ) and decreased the compliance C to account for the stiffening of the arterial segment due to the device implantation. In all cases, the treatment of an aneurysm in the left ICA was simulated. A maximum relative change in flow resistance and inertance of 30% (see below), and a 10× decrease in compliance were studied as they were considered to represent worst-case scenarios, corresponding in general to large aneurysms with strong inflows.

A total of 896 scenarios were considered: 16 circle of Willis configurations, seven levels of collateralization (excellent to none), four aneurysm characteristics (increase/decrease of resistance and inertance), and 2 lengths of vascular rigidization due to stent implantation (stent length and twice the stent length). Simulations were run for each of these scenarios and the change in pressure, pressure pulsatility (defined as max minus min during cardiac cycle), and wall shear stress (WSS), from the pre-to the post-treatment were computed along the entire network.

## 3 Results

### 3.1 Local changes of aneurysmal segment

The analysis of the 27 patient-specific aneurysm geometries (see Supplementary Appendix S6) revealed that removal of the aneurysm by a FD device resulted in a change of the local arterial resistance of approximately |ΔR|=12.5±15.6% , and a change in the segment inertance of approximately |ΔL|=15.6±19% . Moreover, results showed a linear correlation between ΔR and ΔL (*R* ^2^ = 0.91, *p* < 0.001). There was no clear association between these changes and aneurysm size or shape, however the maximum changes seemed to correspond to aneurysms with inertia driven flows, i.e. with strong inflow jets entering the aneurysm before removal that were effectively redirected away from the aneurysm by the flow diverter. Two representative examples of aneurysms with shear and inertia driven flows are presented in Supplementary Appendix S6, Supplementary Figure S9).

### 3.2 Distal pressure

**FIGURE 3:**
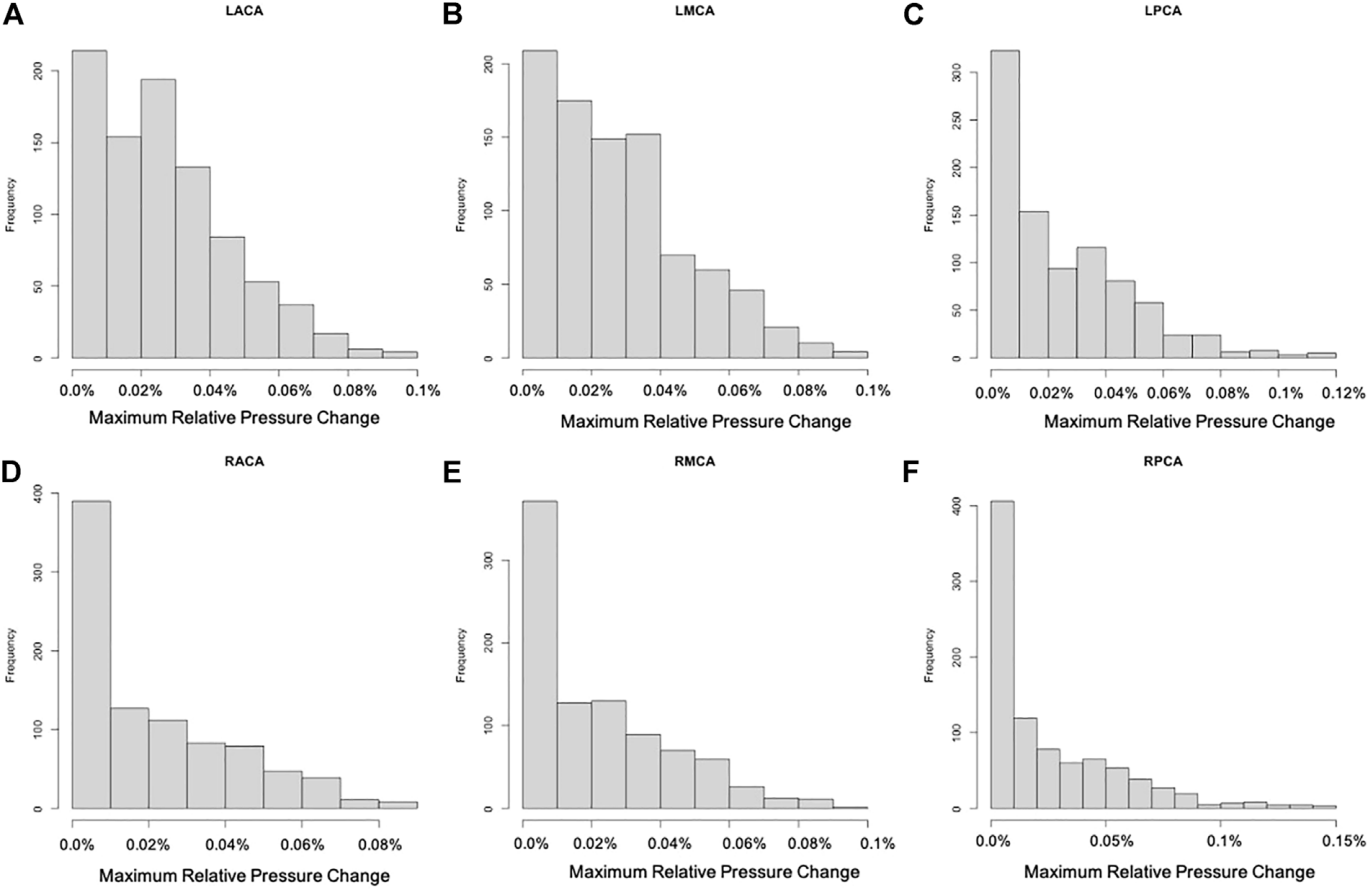
Histograms showing the frequency of maximum relative pressure change for each of the six cerebral arterial trees obtained for the different scenarios considered: **(A)** LACA, **(B)** LMCA, **(C)** LPCA, **(D)** RACA, **(E)** RMCA, **(F)** RPCA. These histograms show that the largest change was below 0.15% (maximum change occurred in the posterior cerebral artery trees).

**FIGURE 4:**
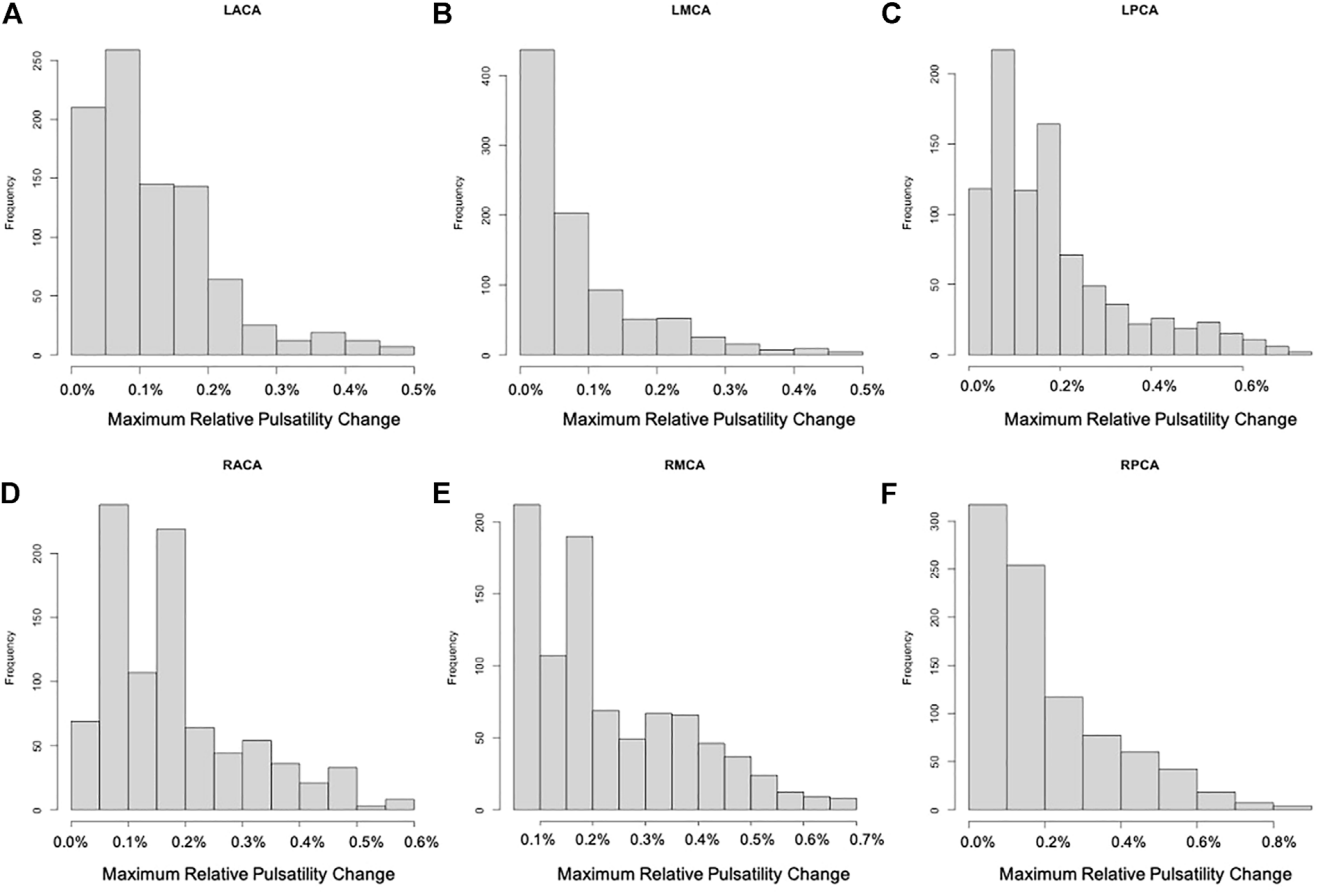
Histograms showing the frequency of maximum relative change of pulsatility for each of the six cerebral arterial trees obtained for the different scenarios considered: **(A)** LACA, **(B)** LMCA, **(C)** LPCA, **(D)** RACA, **(E)** RMCA, **(F)** RPCA. These histograms show that the largest change was approximately 0.85% (maximum change occurred in the posterior cerebral artery trees).

For each of the 896 scenarios considered, the maximum relative change of the pressure and pulsatility over the entire vascular network were calculated. Histograms of frequency of these maximum changes for each of the six cerebral arterial trees were calculated and are presented in Figures 3, 4, respectively. It can be seen that the changes in pressure were very small, below 0.15% for all scenarios. Similarly, the changes in pulsatility were also minimal, below 0.85% for all scenarios.

### 3.3 Flow reversal in distal collaterals

**FIGURE 5:**
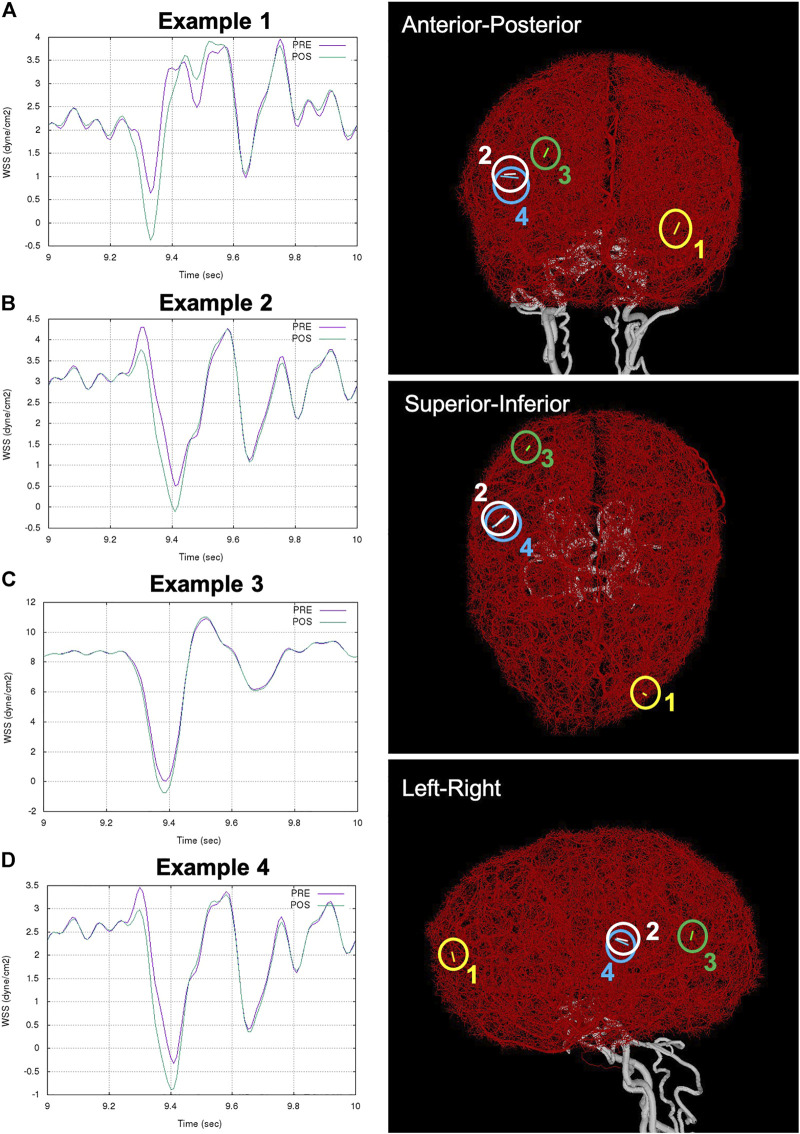
Examples of flow reversal in distal collaterals: **(A)** Example 1: WSS in collateral between left MCA and ACA trees (ipsilateral to aneurysm and FD), **(B)** Example 2: WSS in collateral between right MCA and PCA trees (contralateral to aneurysm and FD), **(C)** Example 3: WSS reversal in collateral between right MCA and PCA trees, and **(D)** Example 4: accentuation of flow reversal in collateral between right MCA and PCA. Anatomical location of the example collaterals (marked with circles and numbered) are shown on the right column in three orthogonal views.

Next, we searched for possible new flow reversals induced in the different arterial networks by the FD implantation. For this purpose, arterial branches in which the WSS changed sign during the cardiac cycle were identified and analyzed. Representative examples are presented in Figure 5. The left column of this figure shows the variation of WSS in four example collaterals during the last cardiac cycle considered. The right column shows the anatomical location of these small collateral branches in three orthogonal views.

Example 1 shows a new flow reversal that was created after FD deployment in a collateral vessel connecting the left MCA and the left ACA trees (i.e. ipsilateral to the treated aneurysm). This case corresponded to circle of Willis variant number 15 and very good distal collateralization. Figure 5A shows the WSS in this branch during the cardiac cycle. Before treatment, the WSS range was [ $0.64,\;3.96\;dyne/cm^{2}]$ , while after treatment it became [ $-0.37,\;3.91\;dyne/cm^{2}]$ .

Example 2 shows another case with a new flow reversal in a collateral connecting the right MCA and right PCA trees (i.e. contralateral to the treated aneurysm. This case corresponded to circle of Willis variant number 7 and poor distal collateralization. In this case (Figure 5B), the WSS range changed from $\left[0.51,\;4.30\;dyne/cm^{2}\right]$ to $\left[-0.1,\;4.26\;dyne/cm^{2}\right]$ .

Example 3 shows a collateral with a new flow reversal. This collateral connects the right MCA and the right PCA, and the WSS range changed from [0.05,10.8 $dyne/cm^{2}$ ] to [-0.75,11.0 $dyne/cm^{2}$ ] (Figure 5C). This case corresponded to the circle of Willis variant number 7 and very good distal collateralization. In this same scenario, example 4 (Figure 5D) shows the accentuation of a small flow reversal already present in a collateral before treatment. The WSS range in this collateral, which also connected the right MCA and right PCA, changed from [-0.33,3.46 $dyne/cm^{2}$ ] to [-0.88,3.31 $dyne/cm^{2}$ ].

Finally, it must be noted that this kind of flow reversal in collateral branches seems to be quite rare, they were detected in less than 4% of the scenarios considered, and only in a handful of collateral vessels connecting different arterial trees.

## 4 Discussion

Delayed intra-parenchymal hemorrhage following flow diversion treatment of intracranial aneurysms continues to be an unexplained and grave complication. In this study, we employed a detailed computational model of the brain arterial network that includes both primary collaterals in the circle of Willis as well as small distal collaterals connecting the different arterial trees perfusing each hemisphere to study the hemodynamic effects in small distal vessels caused by the FD treatment of a proximal intracranial aneurysm.

The model predicted a very small increase of the intra-arterial pressure or its pulsatility (less than 1%) in the small distal vessels after FD treatment of an ICA aneurysm. This small pressure increase, which is primarily due to the change in resistance caused by the exclusion of the aneurysm by the FD, is likely to be compensated by autoregulation and is not expected to cause any important complication or to provide a possible explanation for the DIPHs. This finding agrees with results from a previous study using a much simpler three compartment Windkessel model (Mitha et al., 2015).

On the other hand, we observed that for particular configurations of the circle of Willis and levels of distal collateralization the model predicted that by implanting a FD to treat an ICA aneurysm it is possible to induce new flow reversals in small collateral vessels. This effect is due to the de-phasing of pressure waveforms traveling at slightly different speeds along the arterial network caused by local changes in the compliance of the aneurysmal segment after implantation of the FD (and to a lesser extent due to the change in inertance caused by the exclusion of the aneurysm).

Previous experimental studies have investigated the effects of flow reversal on endothelial cells (Dessalles et al., 2021). One study showed that flow reversal resulted in an important increase of NO production, which significantly influences the transport properties of the endothelium (Hillsley and Tarbell, 2002). A later study demonstrated upregulation of pro-inflammatory markers and gene expression that resulted in a pro-atherogenic response of endothelial cells (Amaya et al., 2016). Another study showed that the inability of endothelial cells to align in low and oscillatory flows led to inflammatory activation (Wang et al., 2013), while a subsequent study showed that low and reversing wall shear stress induced inflammatory responses in endothelial cells (Dabagh et al., 2017). Yet another study showed that flow reversal induced adhesion of monocytes which differentiated into macrophages (Conway and Schwartz, 2013). These observations support the idea that flow reversal may induce endothelial dysfunction and have a detrimental effect on the vascular wall through an inflammatory process.

Therefore, we would like to propose the hypothesis that flow reversal in small collateral vessels induced by wave propagation effects in complex arterial networks after FD treatment of intracranial aneurysms as a potential mechanism to explain DIPHs. Interestingly this hypothesis is consistent with several observations related to DIPH. First, since this is a biological mechanism (rather than mechanical), it is expected to occur in a delayed fashion, not immediately. Second, depending on the configuration of the circle of Willis and collateralization of the brain, flow reversals (and therefore distal hemorrhages) can occur not only ipsilaterally but also contralaterally. Third, the flow reversals are observed mostly on small collaterals, consistent with a distal origin of the hemorrhages. Fourth, the proposed mechanism is not affected if coils are used in combination with flow diverters for the aneurysm treatment. Finally, flow reversals are rare (they were observed in less than 4% of the scenarios considered and for only a few collaterals), they only occur for particular combinations of circle of Willis variants, levels of collateralization, aneurysm characteristics associated with larger changes in flow inertance and resistance, and flow diverters that rigidize the parent artery. Further studies would be required to determine the expected frequency of occurrence of these flow reversals in the actual aneurysm patient population (i.e. using the frequency of occurrence of the anatomical configurations considered in this study). Additionally, it has been noticed that DIPH seems to occur frequently in giant aneurysms and several of them were treated with multiple devices. Giant aneurysms are more likely to exhibit an inertia driven flow with a strong inflow jet (see Supplementary Figure S9), which would produce larger alterations of inertance and resistance when excluded from the circulation by the FD, and using multiple devices could also produce a larger alteration of the compliance of the treated segment. These effects could increase the chances of DIPH for this type of aneurysms. If our hypothesis is confirmed in future studies, computational models could be made patient-specific and used to identify patients and aneurysms at higher risk of developing DIPH after FD aneurysm treatment and managed differently or treated by some other surgical technique.

Our study has several limitations. Parameters of the CCO algorithm used to extend the arterial trees beyond what is visible in the MRA images could be changed to generate arterial trees with different local anatomical characteristics. Other anatomical characteristics of the distal collateralization, such as anterior/posterior dominance, or left/right hemispheric imbalance, etc. Could be considered. It is not known how small patient-specific local anatomical details could alter the flow results. Our models did not account for possible arteriogenesis or pruning of the collateral vessels (Faber et al., 2014). These processes usually occur in response to large increases in flow or wall shear stress associated with vessel ligations or occlusions (Della-Morte and Rundek, 2015; Nishijima et al., 2015). The flow changes due to aneurysm treatment analyzed here are typically much smaller than in those situations. Additionally, the time scales of these processes may range from days to several weeks (Buschmann and Schaper, 2000), while DIPH occurs between 1 week and 1 month, but the expression of pro-inflammatory signals by endothelial cells exposed to flow reversal occurs in hours (Buschmann and Schaper, 2000). Arteriogenesis occurs in response to high changes in flow, which is not the case for FD treatment of aneurysms. The time scales for arteriogenesis and DIPH do not completely overlap. However, expressions of pro-inflammatory signals are observed a few hours after exposure to reversing flow. Thus, it is expected that the flow reversals created immediately after FD implantation could potentially have a negative effect on the subsequent structure and integrity of collateral vessels and be responsible for DIPHs. The flow model also made several simplifying assumptions: Newtonian flow, elastic walls, no autoregulation, etc. As in several previous studies (Peiro and Veneziani, 2009; Reymond et al., 2009; Obeid et al., 2022), wall thickness was estimated as a fraction of the vessel diameter and elastic modulus was approximated as constant throughout the network (including collaterals). The impact of all these assumptions and approximations on the calculated hemodynamic quantities requires a comprehensive analysis and will be the subject of future studies. Finally, the flow reversals identified in our studies had small amplitudes and lasted for a short portion of the cardiac cycle, therefore it is not clear if the effects of flow reversal on endothelial cells observed in experiments (Wang et al., 2013; Dabagh et al., 2017) would be sufficient to predispose collaterals to hemorrhage. Nevertheless, despite these limitations, the models described in this paper allowed us to suggest an intriguing hypothesis for the mechanism of DIPH that does not contradict any of the known facts about DIPHs and that should be further investigated in future studies.

## 5 Conclusion

Delayed intra-parenchymal hemorrhage after flow diversion treatment of intracranial aneurysms remains a rare but serious and unexplained complication. A detailed computational model of the brain arterial network predicted, for particular combinations of circle of Willis configuration and level of distal collateralization, dynamic flow reversal in small collaterals after implantation of flow diverters to treat intracranial aneurysms. These flow reversals during the cardiac cycle could have a detrimental effect on the vessel wall *via* inflammatory processes and could provide a plausible mechanism for distal hemorrhages after flow diversion.

## Data Availability

The raw data supporting the conclusion of this article will be made available by the authors, without undue reservation.
