# Supplementary material for: Flow reversal in distal collaterals as a possible mechanism of delayed intraparenchymal hemorrhage after flow diversion treatment of cerebral aneurysms
Source: Front Physiol. 2022 Jul 18;13:881627. doi: 10.3389/fphys.2022.881627 (PMC9339966; doi:10.3389/fphys.2022.881627)
Supplement: Supplementary file 1 [file DataSheet1.pdf]

SUPPLEMENTARY MATERIAL

Supplementary Appendix 1

Arterial Tree Extension and Generation of Pial Collaterals

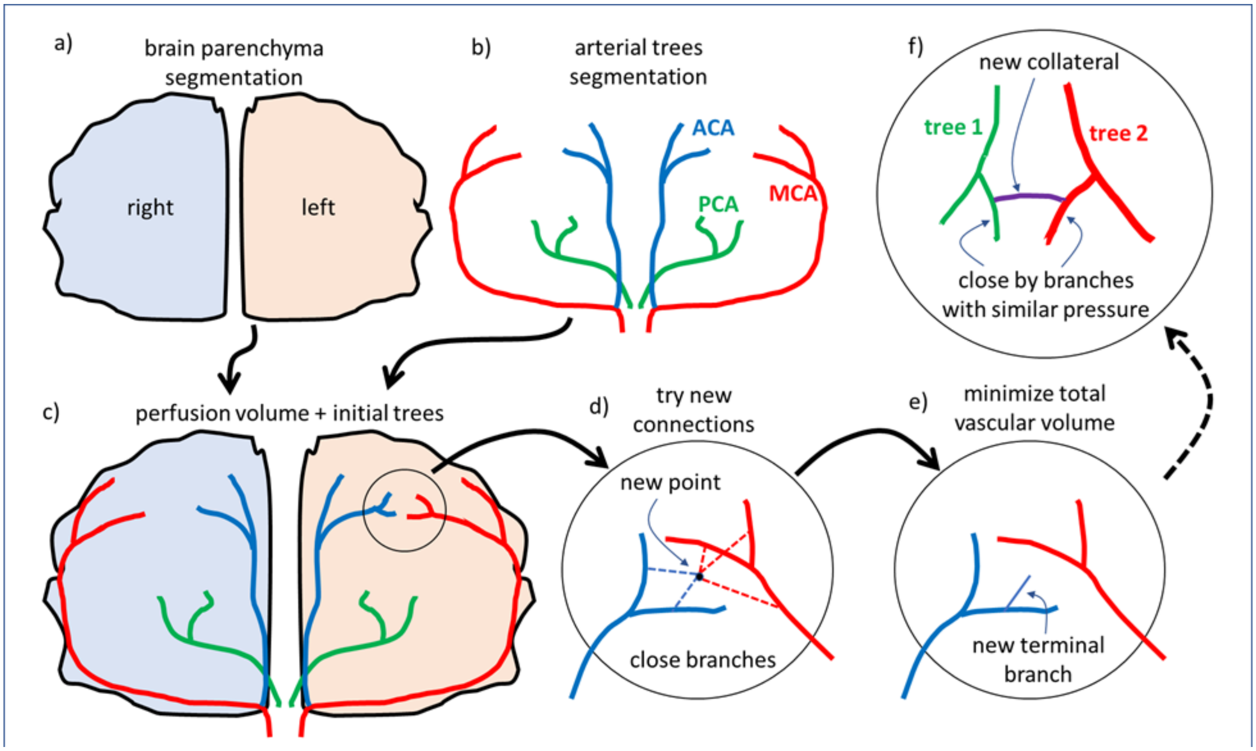

Supplementary Figure I. *Creation of vascular model: a) rough segmentation of the brain parenchyma and meshing with tetrahedral element of the left and right hemispheres, b) reconstruction of arterial trees from MRA images, c) initialization of CCO algorithm with hemisphere brain parenchyma (perfusion volume) and vascular reconstructions (initial arterial trees), d) try new connections from randomly selected point in perfusion volume to nearby branches of the different arterial trees, rescaling the downstream trees to accommodate the flow through the new branch, e) form new terminal branch that minimizes the total vascular volume and does not intersect any other branch, and f) once the trees have been extended, generate pial collaterals by connecting nearby branches of different trees within a certain diameter range and that have similar pressures to ensure no or little flow through the collateral under normal conditions.*

Supplementary Appendix 2

Variants of the Circle of Willis

Different anatomical variants of the circle of Willis were created by alternatively occluding the posterior and anterior communicating arteries, as well as the A1 segments of the anterior cerebral arteries. The different configurations considered are summarized in Supplementary Table I, and illustrated in Supplementary Figure II.

Variant No.	Posterior communications		Anterior communications		
	L-PCOM	R-PCOM	ACOM	L-A1	R-A1
1	+	+	+	+	+
2	+	+	+	+	-
3	+	+	+	-	+
4	+	+	-	+	+
5	+	-	+	+	+
6	+	-	+	+	-
7	+	-	+	-	+
8	+	-	-	+	+
9	-	+	+	+	+
10	-	+	+	+	-
11	-	+	+	-	+
12	-	+	-	+	+
13	-	-	+	+	+
14	-	-	+	+	-
15	-	-	+	-	+
16	-	-	-	+	+

Supplementary Table I. *Variants of the circle of Willis. "+" indicates open artery, "-" indicates occluded (i.e. excluded) artery. See Supplementary Figure II for abbreviations and schematic illustration of the circle of Willis.*

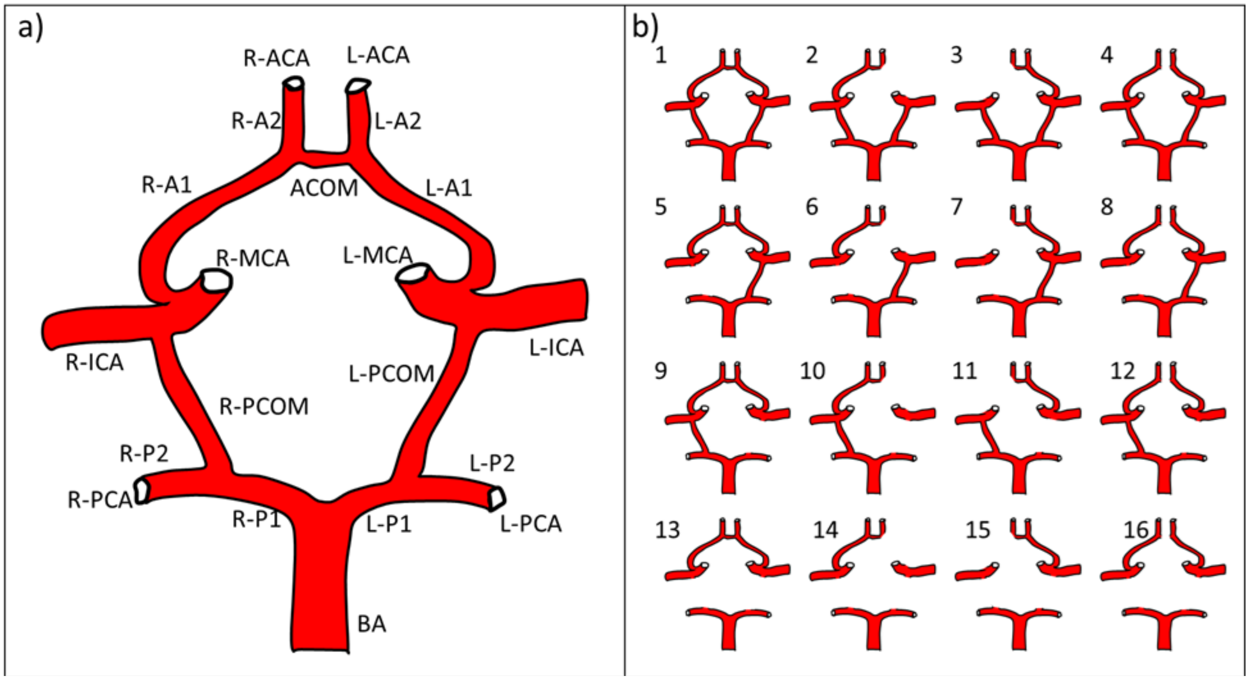

Supplementary Figure II: *a) Circle of Willis, b) anatomical variants considered in this study (numbers correspond to variant numbers listed in Supplementary Table I). Abbreviations: L=left, R=right, ACA=anterior cerebral artery, MCA=middle cerebral artery, PCA=posterior cerebral artery, ICA=internal carotid artery, BA=basilar artery, ACOM=anterior communicating artery, PCOM=posterior communicating artery, A1,A2=segments of the ACA, P1,P2=segments of the PCA.*

Supplementary Appendix 3

Distributed Lumped Parameter Model

The arterial network is subdivided into a series of interconnected compartment. Each compartment corresponds to a straight cylindrical segment of length l, undeformed radius r_0, and wall thickness h (see Supplementary Figure IIIa). The lumped parameter model takes into account, for each compartment, the flow resistance R due to blood viscosity, the inertance L due to blood inertia, and the arterial wall compliance C. These parameters are calculated as:

$$R = \frac{8\mu l}{\pi r_0^4}, \qquad L = \frac{\rho l}{\pi r_0^2}, \qquad C = \frac{3\pi r_0^3 l}{2Eh}$$

where ρ is the blood density, μ is the blood viscosity, and E is the elastic modulus of the wall.

Using the notation indicated in Supplementary Figure IIIb (electrical analog), the governing equations (conservation of mass and momentum) in each compartment can be written as[15]:

$$\begin{cases} L\dfrac{dQ_1}{dt} = P_1 - P_2 - RQ_1 \\ C\dfrac{dP_2}{dt} = Q_1 - Q_2 \end{cases}$$

where P_1 and P_2 are the pressures at the inlet and outlet of the compartment, and Q_1 and Q_2 the corresponding volume flow rates. It has been shown that the solution of these compartment equations converge to the solution of the 1D Navier-Stokes equations as the compartment length tends to zero[15].

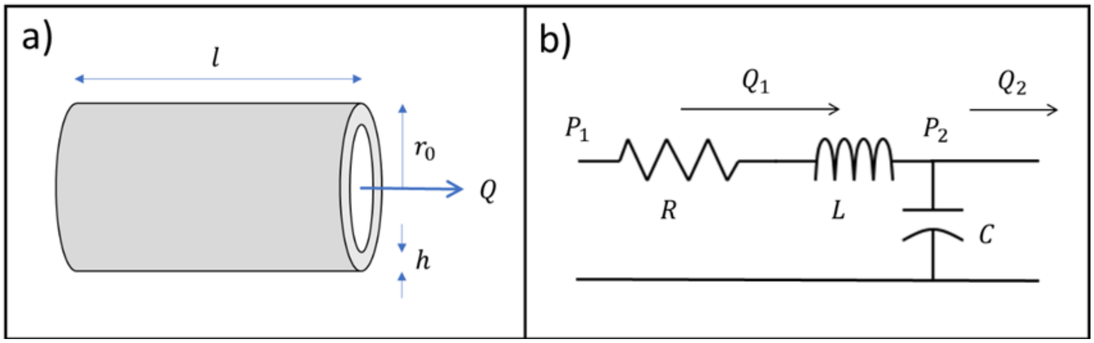

Supplementary Figure III: *a) arterial compartment geometry, b) electric analog for the arterial blood flow lumped parameter model.*

At the network model entrance, i.e. inlets of the left and right common carotid and vertebral arteries, realistic pulsatile flow rates are imposed as boundary conditions (Supplementary Figure IVa). At the model outlets, resistance boundary conditions are prescribed that produce flow divisions consistent with Murray's law. The model thus produced realistic pressure waveforms at the inlets, as shown in Supplementary Figure IVb.

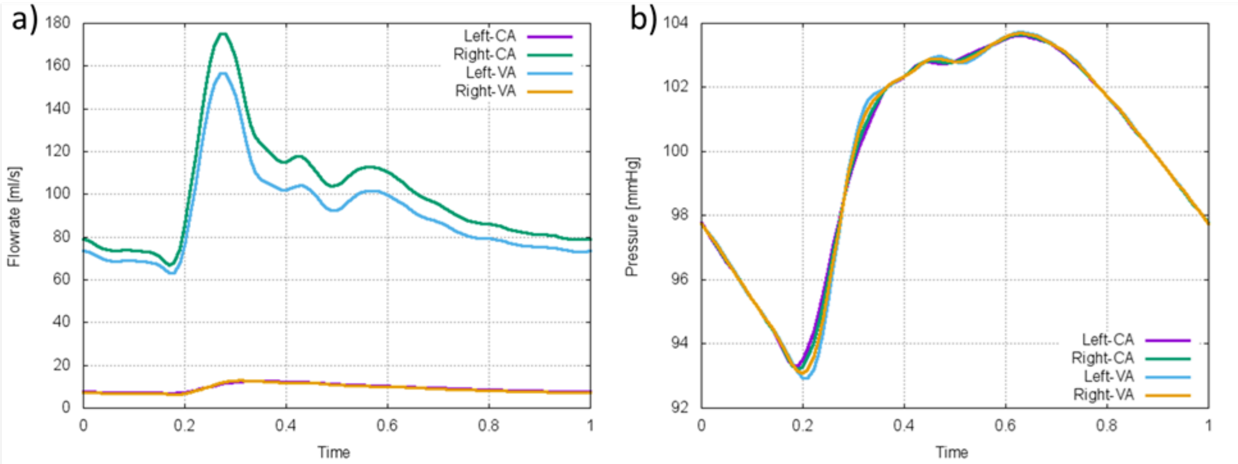

Supplementary Figure IV: *a) realistic pulsatile flow rates imposed as inlet boundary conditions at the origins of the left and right common carotid arteries and the left and right vertebral arteries, b) pressure waveforms obtained with the network model at the origin of these arteries.*

The governing equations were discretized in time using a backward Euler method which results in the following fully implicit scheme:

$$(L + \Delta t R)Q_1^{n+1} + \Delta t P_2^{n+1} - \Delta t P_1^{n+1} = LQ_1^n$$

$$CP_2^{n+1} + \Delta t Q_2^{n+1} - \Delta t Q_1^{n+1} = CP_2^n$$

where n indicates the timestep and Δt the timestep size. The resulting system of linear equations was solved using the UMFPACK solver of the Suite Sparse[16]. All simulations were run for 10 cardiac cycles to eliminate initial condition effects, and the results of the last cycle were used for further analysis (it was verified that the solution did not significantly change from cycle 9 to 10)

Once the flow in each compartment has been found, the wall shear stress is computed assuming fully developed flow and parabolic profile (i.e. Poiseuille's formula):

$$\tau = \frac{4\mu Q}{\pi r^3}$$

The convergence of the solution with respect to the timestep size was analyzed by running simulations with different step sizes in the range $\Delta t = 0.01 - 0.00001\ s$ and identifying the step size beyond which the solution and in particular the wall shear stress was essentially unchanged. An example is shown in Supplementary Figure V where the pressure and wall shear stress in a collateral branch as a function of time is plotted for different timestep sizes. Based on this result a timestep of $\Delta t = 0.0001\ s$ was selected for the study.

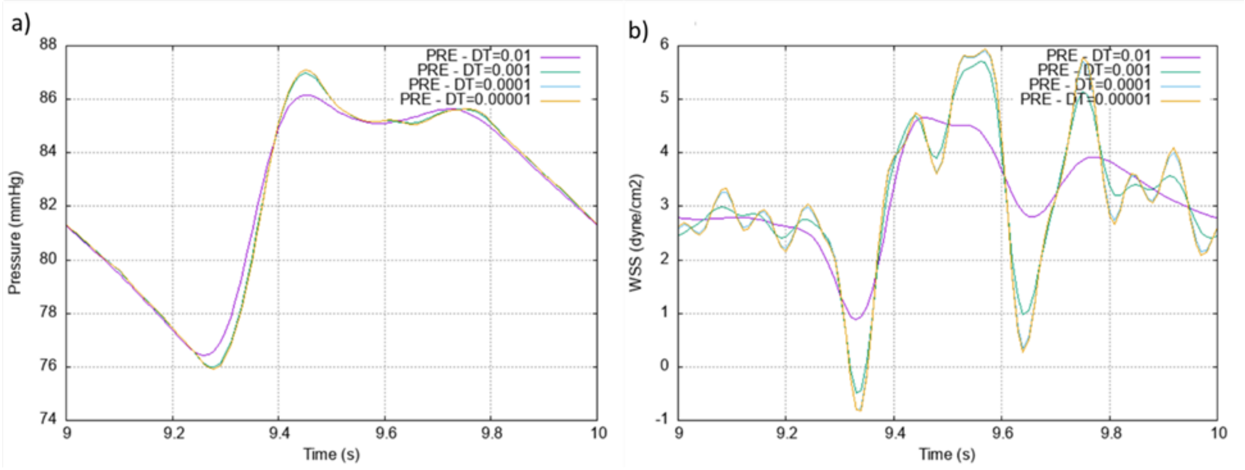

Supplementary Figure V: *Effects of timestep size on time-dependent hemodynamic variables in a collateral vessel: a) pressure, b) wall shear stress magnitude. The solution is essentially unchanged for* $\Delta t = 0.0001s$ *and smaller.*

Supplementary Appendix 4

Estimating Changes of Flow Resistance Caused by the Presence of an Aneurysm

The change of the hydraulic resistance of the parent artery segment caused by the presence of an aneurysm, was estimated from steady CFD simulations performed on 27 patient-specific aneurysm geometries. To perform these CFD simulations, vascular models were generated from vessel lumen segmentations of 3D rotational angiography images and then the 3D models were used to generate unstructured computational meshes composed of tetrahedral elements employing an advancing front method [15]. The maximum element size was 0.2 mm, resulting in meshes ranging from 2 to 4 million elements. The incompressible Navier-Stokes equations were used to model the blood flow with viscosity of 0.04 Poise and density of 1g/cm^3.An in-house fully implicit finite element code was used to numerically solve these equations [22]. Velocity boundary conditions were prescribed at the inlets using the Womersley velocity profile and mean flow rates obtained from an empirical flow-area relationship [23]. At the outlet, zero pressure boundary conditions were prescribed. Vessel walls were approximated as rigid and no-slip boundary conditions were prescribed at the walls. In each case, two models were considered, one the original model containing the aneurysm, and a second model in which the aneurysm was virtually removed to simulate the conditions without the aneurysm.

For each aneurysm geometry, the skeleton of the parent artery was constructed and points proximal and distal from the aneurysm segment were identified, as illustrated in Supplementary Figure VI.

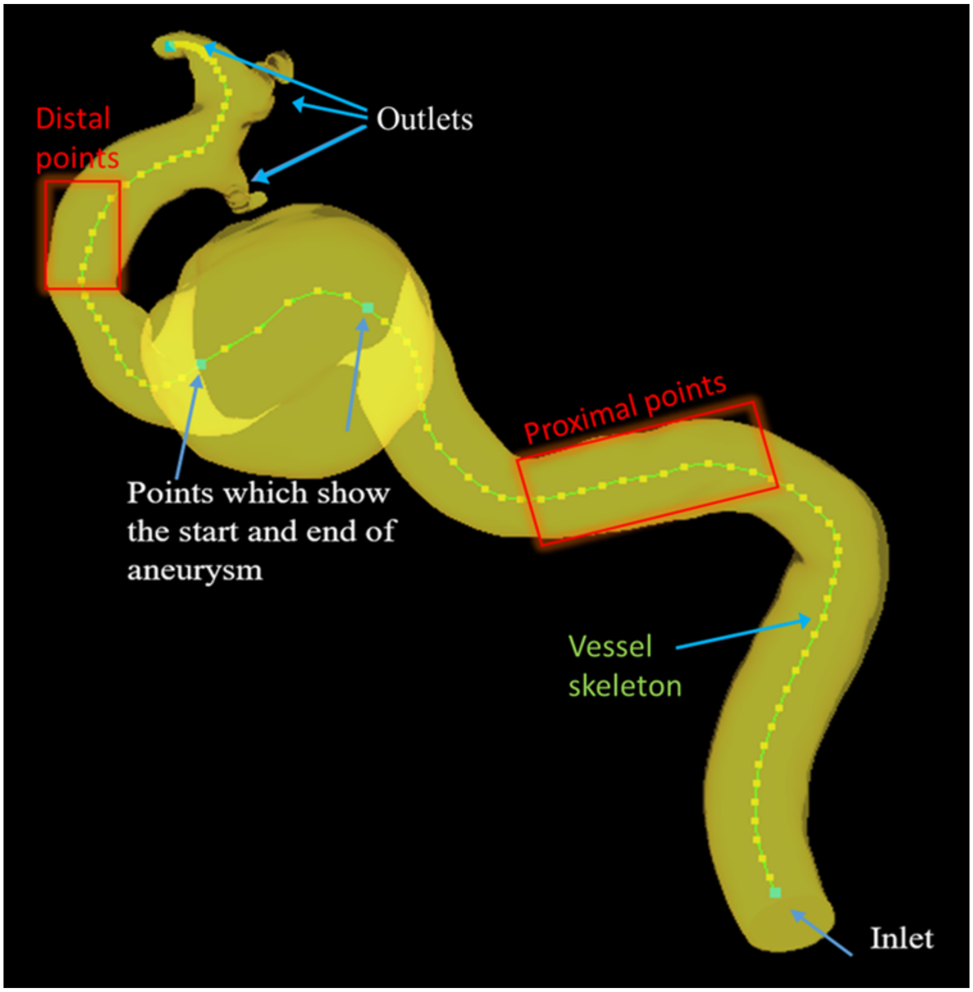

Supplementary Figure VI: *Computing pressures along parent artery skeleton to estimate changes in hydraulic resistance of aneurysmal segment after treatment with FD device.*

Three steady state CFD simulations were carried out corresponding to arbitrary but realistic "high", "medium" and "low" inflow rates, and the pressure drop from the proximal to the distal points was calculated for each inflow rate for the original model (containing the aneurysm) and the model with the virtually removed aneurysm. An example is presented in Supplementary Figure VIIa, where the pressure along the parent artery skeleton is plotted for the high (black), medium (red) and low (blue) flow condition, for both the original model with the aneurysm (solid lines) and the models with the virtually removed aneurysm (dashed lines). The aneurysmal segment is indicated by the ovals. The pressure drop across the aneurysmal segment is plotted against the inflow rate in Supplementary Figure VIIb for one of these cases (virtually removed aneurysm). The slope of this curve gives the hydraulic resistance of the aneurysmal segment: $\Delta P = R \cdot Q$.

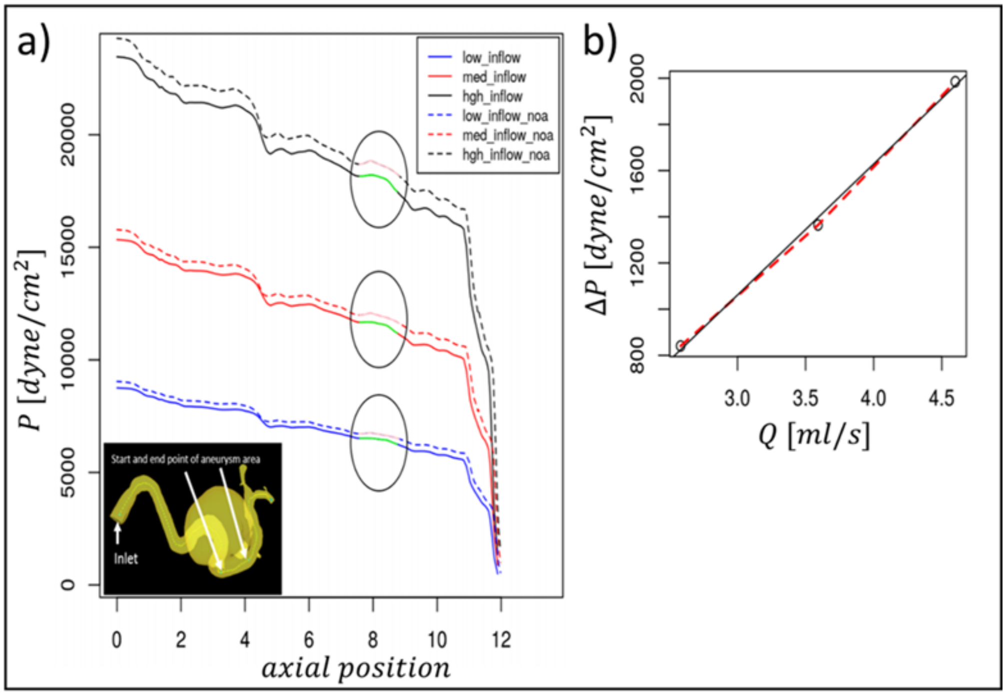

Supplementary Figure VII: *a) pressure along vessel skeleton for three inflow rates and for the model with and without the aneurysm; b) pressure drop (ΔP) vs. inflow rate (Q) for the case without aneurysm, the slope of this curve gives the hydraulic resistance R.*

Thus, the percent relative change in the hydraulic resistance of the aneurysmal segment due to the presence of the aneurysm was calculated as:

$$\Delta R = \frac{R_{without} - R_{original}}{R_{original}} \times 100$$

where $R_{without}$ is the resistance of the model with the aneurysm virtually removed, and $R_{original}$ is the resistance of the original model. The process was repeated for the 27 patient-specific geometries and the maximum resistance change was calculated.

Supplementary Appendix 5

Estimating Changes of Flow Inertance Caused by the Presence of an Aneurysm

The change of the local inertance of the parent artery due to the presence of a cerebral aneurysm was estimated by performing unsteady CFD simulations (more details about CFD simulations has been described in the first paragraph of Appendix 4) with a sinusoidal flow waveform as the inlet boundary condition. Specifically, the inflow rate was prescribed as (see Supplementary Figure VIIIa):

$$Q(t) = Q_0 + q\, e^{i\omega t}$$

where Q_0 is a mean steady state flow rate, q is the amplitude of the sinusoidal flow waveform, ω is the (angular) frequency and t is the time. Similarly, the pressure drop in the aneurysmal segment can be separated into steady and unsteady contributions:

$$\Delta P(t) = \Delta P_0 + p\, e^{i\omega t}$$

where ΔP_0 is the mean steady state pressure drop, and p is the amplitude of the pressure waveform. Inserting these forms for the flow rate and pressure into the lumped parameter equation

$$\Delta P = R \cdot Q + L \frac{dQ}{dt}$$

the first term gives the mean pressure drop:

$$\Delta P_0 = R \cdot Q_0$$

while the second term gives the unsteady pressure waveform:

$$p\, e^{i\omega t} = R \cdot q\, e^{i\omega t} + L \cdot i\omega q\, e^{i\omega t}$$

$$\Longrightarrow \Delta P_L = p - R \cdot q$$

Therefore, the inertance L is obtained as the slope of the ΔP_L vs. ωq plot obtained by running 3 CFD simulations with varying flow amplitudes (q) and using the values of R obtained as explained in Supplementary Appendix 4. An example is shown in Supplementary Figure VIIIb.

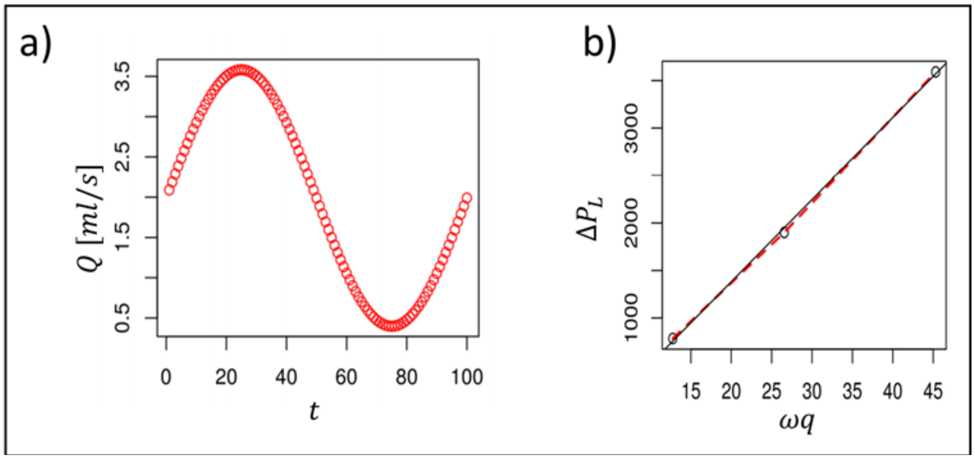

Supplementary Figure VIII. *a) example sinusoidal inflow rate waveform; b) example pressure wave amplitude (q) vs. frequency times flow amplitude (ωq), the slope of this curve gives the inertance L.*

As with the change in resistance, the percent relative change in the aneurysmal segment inertance due to the presence of the aneurysm was calculated as:

$$\Delta L = \frac{L_{without} - L_{original}}{L_{original}} \times 100$$

where $L_{without}$ is the inertance of the model with the aneurysm virtually removed, and $L_{original}$ is the inertance of the original model. The process was repeated for the 27 patient-specific geometries and the maximum inertance change was calculated.

Supplementary Appendix 6

Estimated changes in flow resistance and inertance

The changes in hydraulic resistance and inertance of a vessel segment due to the presence of an aneurysm were calculated as explained in Supplementary Appendix 4 and 5. For this purpose, the patient-specific vascular geometries of 27 patients with aneurysms in the internal carotid artery (ICA) were studied. Supplementary Table II presents the distribution of aneurysm location, aneurysm size, neck size, aspect ratio along with the mean aneurysm inflow rate and the change in resistance and inertance caused by the presence of the aneurysm for the 27 cases studied.

Aneurysm	Location	Asize (cm)	Nsize (cm)	AR	Q (ml/s)	Q/Inflow(%)	ΔR (%)	ΔL (%)
1	ICA-oph	2.29	1.62	1.13	5.70	100.0	-24.4	-36.8
2	ICA-cav	0.71	0.58	0.54	0.76	100.0	11.0	9.5
3	ICA-oph	1.70	1.24	1.04	3.53	100.0	22.5	16.6
4	ICA-pcom	0.97	0.68	0.92	1.15	100.0	17.1	19.4
5	ICA-pcom	0.52	0.30	1.20	0.41	100.0	20.9	20.6
6	ICA-pcom	0.87	0.64	0.87	1.07	100.0	25.2	27.5
7	ICA-cav	1.65	1.00	1.19	1.36	56.3	11.3	27.2
8	ICA-cav	0.76	0.51	1.07	0.83	41.5	-3.8	-5.8
9	ICA-cav	1.82	0.85	1.87	1.65	83.1	7.5	13.0
10	ICA-cav	0.30	0.30	0.28	0.20	5.1	-1.6	-5.6
11	ICA-shyp	0.39	0.33	0.78	0.20	6.8	-8.7	-12.4
12	ICA-cav	1.99	1.48	0.81	2.75	64.5	4.8	4.2
13	ICA-cav	3.54	0.51	2.48	1.69	56.2	3.8	3.1
14	ICA-cav	0.38	0.24	1.01	0.04	2.1	-3.6	-16.7
15	ICA-pcom	0.50	0.44	0.54	0.54	27.1	14.2	17.0
16	ICA-oph	0.39	0.36	0.68	0.23	11.1	6.9	9.1
17	ICA-cav	2.60	1.61	1.50	3.53	159.2	-51.3	-49.9
18	ICA-cav	2.65	1.24	1.65	1.56	56.6	5.5	18.0
19	ICA-cav	0.77	0.94	0.49	3.26	100.0	1.7	-7.8
20	ICA-oph	0.66	0.48	0.80	0.29	14.4	-6.9	-7.7
21	ICA-cav	1.59	1.10	0.75	2.95	83.7	9.3	20.0
22	ICA-shyp	2.26	0.93	1.99	1.97	98.4	-5.9	-1.7
23	ICA-oph	1.08	0.62	0.95	1.64	56.5	12.9	10.0
24	ICA-shyp	0.86	0.52	1.18	0.58	29.1	16.3	10.3
25	ICA-shyp	0.98	0.67	1.03	1.06	53.2	-8.5	-32.1
26	ICA-oph	0.94	0.82	0.51	1.57	77.4	18.2	6.5
27	ICA-cav	1.70	0.80	1.38	2.32	40.1	13.7	14.1

Supplementary Table II. *Changes of resistance and inertance due to presence of aneurysm in 27 patient-specific vascular geometries with aneurysms in the internal carotid artery. ICA=internal carotid artery, oph=ophthalmic segment, cav=cavernous segment, pcom=origin of posterior communicating artery, shyp=superior hypophyseal segment. Asize=aneurysm maximum size, Nsize=neck maximum size, AR=aspect ratio (aneurysm depth over aneurysm neck size), Q=mean aneurysm inflow rate,* $Q/Inflow$*=percent of parent artery flow entering aneurysm,* ΔR*=change of resistance,* ΔL*=change of inertance.*

Examples of two aneurysms, one with a “shear driven” flow and another with an “inertia driven” flow are presented in Supplementary Figure IX. In the first case (aneurysm 11), the blood stream enters the aneurysm in an almost tangential manner causing reductions (negative changes) in the resistance and inertance of the affected vascular segment. On the other hand, the second case (aneurysm 4) illustrates an inertia dominated flow with a strong inflow jet directly entering the aneurysm from the parent artery and causing an increase (positive changes) in the resistance and inertance of the vascular segment. These examples illustrate that the presence of an aneurysm can induce both increases or decreases in the parent artery resistance and inertance, and Suppl. Table II indicates that the absolute magnitude of typical changes is below approximately 30%.

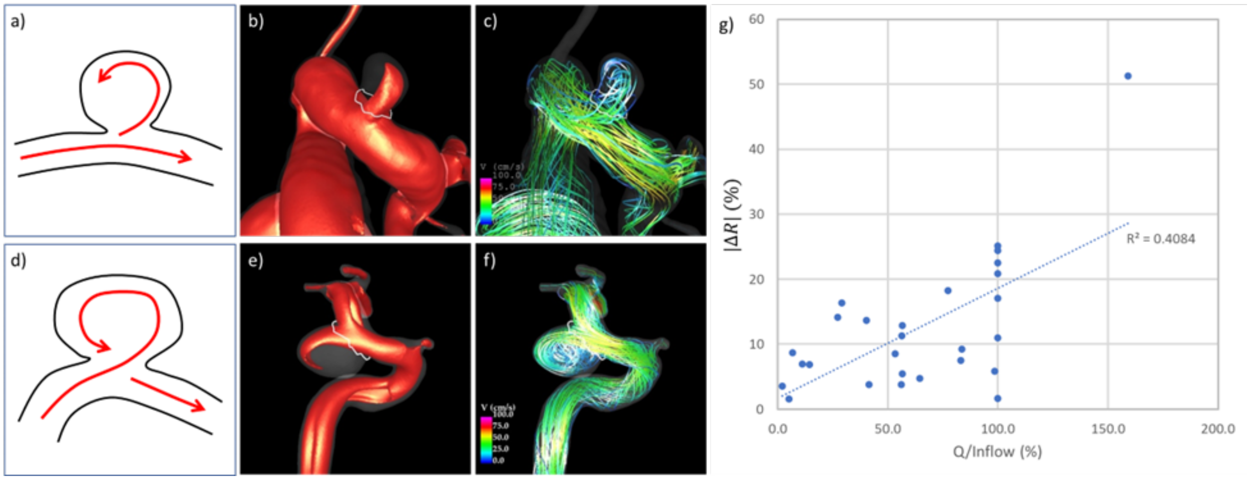

Supplementary Figure IX. *Examples illustrating aneurysms with “shear” and “inertia” driven flows. Top row, shear driven flow (aneurysm 11): a) schematics of shear driven flows, b) aneurysm inflow stream (velocity iso-surface corresponding to* $v = 30\ cm/s$*), c) flow pattern (velocity colored streamlines), Bottom row, inertia driven flow (aneurysm 4): d) schematics of inertia driven flows, e) aneurysm inflow stream (velocity iso-surface corresponding to* $v = 30\ cm/s$*), f) flow pattern, and g) scatter plot of Q/Inflow (% of parent artery flow into the aneurysm) vs magnitude of change in resistance showing a general trend of larger resistance change with higher inertia dominance.*

As observed in Suppl. Figure IXg, one case had a $Q/Inflow$ value larger than 100% (aneurysm 17 in Suppl. Table II). This is caused by recirculating flow in the region of the aneurysm orifice, which makes the blood re-cross the neck surface used to quantify the aneurysm inflow Q and is thus observed as an increased inflow to the aneurysm. As such, this quantity (Q/Inflow) is not a perfect variable to distinguish between inertia and shear driven flows but it provides a rough interpretation of the changes in resistance, and in this particular case the change in resistance was the largest (it decreased by approximately 50%). Visualizations of the flow in this aneurysm are presented in Supplementary Figure X. As can be seen, the entire flow stream from the parent artery enters the aneurysm, and in addition recirculating flow near the aneurism orifice re-enters the aneurysm creating a total inflow rate larger than the flow in the parent artery.

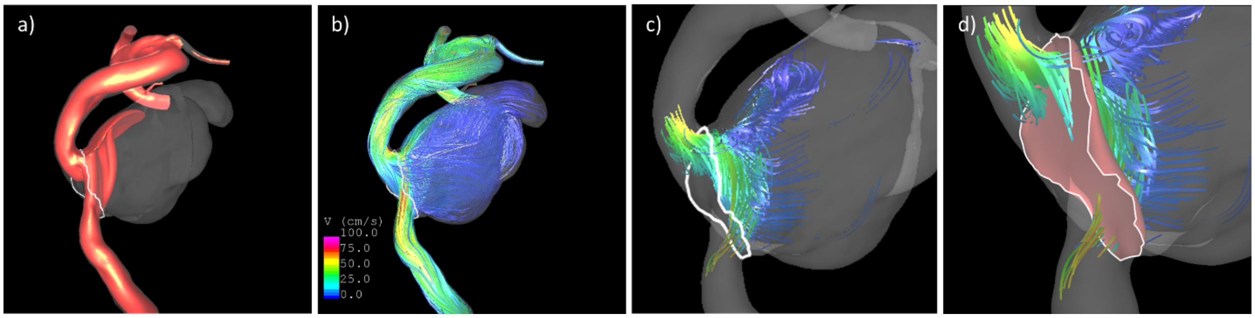

Supplementary Figure X. *Example of inertia driven flow in an aneurysm (number 17 in Table II) with total inflow larger than the flow in the parent artery due to flow recirculation at the aneurysm orifice: a) inflow jet (velocity iso-surface corresponding to* $v = 30\ cm/s$*), b) flow pattern (velocity colored streamlines), c) streamlines illustrating the recirculating flow at the aneurysm orifice, and d) neck surface used to quantify total inflow into the aneurysm.*
